# MRI signal distribution within the intervertebral disc as a biomarker of adolescent idiopathic scoliosis and spondylolisthesis

**DOI:** 10.1186/1471-2474-13-239

**Published:** 2012-12-03

**Authors:** Julien Gervais, Delphine Périé, Stefan Parent, Hubert Labelle, Carl-Eric Aubin

**Affiliations:** 1 Department of Mechanical Engineering, Ecole Polytechnique, Montreal, Canada; 2 Research Center Sainte-Justine University Hospital, Montreal, Canada

**Keywords:** Intervertebral disc degeneration, MRI, Scoliosis, Spondylolisthesis, Histograms, Descriptive statistics

## Abstract

**Background:**

Early stages of scoliosis and spondylolisthesis entail changes in the intervertebral disc (IVD) structure and biochemistry. The current clinical use of MR T2-weighted images is limited to visual inspection. Our hypothesis is that the distribution of the MRI signal intensity within the IVD in T2-weighted images depends on the spinal pathology and on its severity. Therefore, this study aims to develop the AMRSID (analysis of MR signal intensity distribution) method to analyze the 3D distribution of the MR signal intensity within the IVD and to evaluate their sensitivity to scoliosis and spondylolisthesis and their severities.

**Methods:**

This study was realized on 79 adolescents who underwent a MRI acquisition (sagittal T2-weighted images) before their orthopedic or surgical treatment. Five groups were considered: low severity scoliosis (Cobb angle ≤50°), high severity scoliosis (Cobb angles >50°), low severity spondylolisthesis (Meyerding grades I and II), high severity spondylolisthesis (Meyerding grades III, IV and V) and control. The distribution of the MRI signal intensity within the IVD was analyzed using the descriptive statistics of histograms normalized by either cerebrospinal fluid or bone signal intensity, weighted centers and volume ratios. Differences between pathology and severity groups were assessed using one- and two-way ANOVAs.

**Results:**

There were significant (p < 0.05) variations of indices between scoliosis, spondylolithesis and control groups and between low and high severity groups. The cerebrospinal fluid normalization was able to detect differences between healthy and pathologic IVDs whereas the bone normalization, which reflects both bone and IVD health, detected more differences between the severities of these pathologies.

**Conclusions:**

This study proves for the first time that changes in the intervertebral disc, non visible to the naked eye on sagittal T2-weighted MR images of the spine, can be detected from specific indices describing the distribution of the MR signal intensity. Moreover, these indices are able to discriminate between scoliosis and spondylolisthesis and their severities, and provide essential information on the composition and structure of the discs whatever the pathology considered. The AMRSID method may have the potential to complement the current diagnostic tools available in clinics to improve the diagnostic with earlier biomarkers, the prognosis of evolution and the treatment options of scoliosis and spondylolisthesis.

## Background

Spinal deformities such as scoliosis and spondylolisthesis affect both the structural and the biochemical composition of the intervertebral disc (IVD) [1,2] and lead to its degeneration. Adolescent Idiopathic Scoliosis (AIS) is marked by a wedging of the IVD linked with a displacement of the nucleus pulposus and changes in the glycosaminoglycan and water content repartition [3-6]. Spondylolisthesis is characterized by a loss of IVD height [7] and most of the time a disc degeneration [8]. Current radiologic measurements for AIS and spondylolisthesis are the Cobb angles and the Meyerding grades, respectively. However, they are 2D measurements of 3D spinal deformities and in addition they do not provide information on the biological and phenotypical aspects of the pathological discs.

Magnetic resonance imaging (MRI) can be a powerful tool for the diagnosis of spine pathologies. Diminished signal intensity in the IVD and evidence of radial tears in the annulus fibrosus (AF) were highly associated with positive symptoms on discography [9-18]. Changes in the intervertebral disc height, area or volume were quantified from MR images to highlight the effect of daily activities or various loading of the spine [19-22]. The displacements of the IVD components under various movements of the spine were also measured from MR images, highlighting significant correlations between the nucleus zone migration and the flexion-extension movements of the spine [23-25], or correlations between nucleus zone migration and intervertebral disc wedging in scoliosis [6,26]. Thus MRI can potentially help to monitor spine pathologies progression *in vivo*[27-29]. For instance, MRI gives reliable non-invasive 3D images of the IVD [30] allowing longitudinal follow-up studies in spine pathologies and T2-weighted images are used to assess the IVD degeneration [27,30,31]. Analysis of T2-weighted images can help to detect early signs of disc degeneration [4,32] but the current clinical use of T2-weighted images is for the detection of late stages of the spine pathologies [30].

Recent studies further investigated MRI in the spine field with automated diagnosis. Disc herniation was evaluated with high precision of prediction (specificity 99%, sensitivity 93%) [33,34]. These studies were based on active shape modeling (2D), classifiers (2D) and histograms (2D/3D), which were also used as research tools for automated diagnosis of other diseases such as liver metastases [35] or myocardial fibrosis by shape recognition and histogram analysis [36].

Our hypothesis is that the distribution of the MRI signal intensity within the IVD in T2-weighted images depends on the spinal pathology and on its severity, and consequently has the potential to provide essential information on spine pathologies that could not be appreciated by direct visual observation as currently performed in clinics [37]. Therefore, this study aims to develop new tools to analyze the 3D distribution of the MR signal intensity within the IVD and to evaluate their sensitivity to different spinal deformities (scoliosis or spondylolisthesis) and severities.

## Methods

### Subjects’ selection

**Figure 1:**
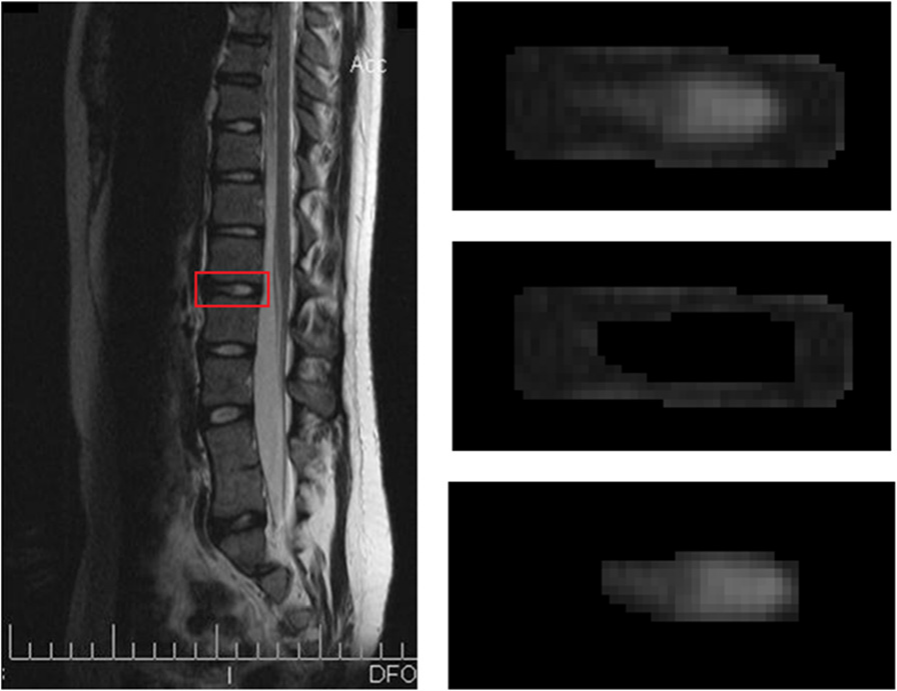
**Segmentation process.** An IVD is selected from a sagittal slice of the whole spine (Left). This IVD is then semi-automatically segmented into three parts: IVD (Top right), AF (middle right) and NP (bottom right).

Seventy-nine subjects (32 with scoliosis, 32 with spondylolisthesis and 15 with herniated IVD) with an average age of 15.1 (range, 8–19.8) were selected from a clinical database of patient’s images. The selected cases presented no other pathologies such as severe discopathies, herniation over the lumbar discs, no brace or surgical treatments prior to the MRI acquisition and/or no artifacts in the images. The subjects were divided into 5 groups: high severity scoliosis (Cobb Angle 50° to 90°, n = 16), low severity scoliosis (Cobb Angle 10° to 49°, n = 16), high severity spondylolisthesis (Meyerding grade III to V, n = 16), low severity spondylolisthesis (Meyerding grade I or II, n = 16) and controls (non-afflicted IVD from herniated patients, n = 15). There were no significant differences in the distribution of the morphological parameters (Table 1) between each group, providing randomization. Each participating subject or in the case of minors, their legal guardian, gave informed consent for the use of their clinical data in future research projects. The research protocol was approved by the research ethics committee of our institutions. MR images (Figure 1) were all performed on the same 1.5 T system (Sonata, Siemens Healthcare, Erlangen, Germany) using a sagittal turbo spin echo T2-weighted sequence (slice thickness 3 mm, gap 3.6 mm, field of view of 350*350 mm, matrix size of 512*512, echo time of 121–126 ms and repetition time of 3,200–3,690 ms).

**Table 1:** Patient morphology and MRI scanning time of the day

**Patient**	**Mean**	**Median**	**Standard deviation**	**Min**	**Max**
Age (years)	15.1	14.8	2.3	8.0	19.8
Height (cm)	161.0	161.7	12.2	108.3	181.6
Weight (kg)	56.3	56.8	13.4	18.3	99.2
Body Mass Index	21.5	21.0	4.1	13.0	36.0
MRI acquisition time of the day	12:10	13:26	1:45	7:40	18:00

### IVD selection and segmentation

For the scoliosis groups, the disc under the apical vertebra of the lumbar curve was selected. For the spondylolisthesis groups, the L4/L5 IVD was selected, as high grades (IV and V) deformation of L5-S1 IVDs gives unreliable MR signal intensity. For the control group, the healthy IVD (L4/L5 or L2/L3) was selected. The segmentation was semi-automatically performed by a M.Sc. student in Biomedical Engineering using Slice-O-Matic (Tomovision, Magog (QC), Canada). This segmentation was realized for three zones (Figure 1): IVD, nucleus pulposus (NP) and annulus fibrosus (AF) and took about fifteen minutes per disc.

### Data normalisation

Two-step normalization was done in order to minimize the discrepancy between the subjects and acquisition techniques (MatLab, Mathworks, Naticks (MA), USA). First, each voxel was set to a fraction of the IVD’s volume to normalize the count of voxels between patients’ histograms due to the variable disc sizes and image resolution between subjects. Then, MR intensity values were divided by normalizing intensities to minimize the variability due to variable patients’ morphologies, variable MR acquisition gain and slightly variable TR and TE parameters. Two different normalizing intensities were used: the mean cerebrospinal fluid (CSF) intensity, which is known for its constant chemical composition and which is already used in the literature to normalize MR intensities [38-40], and the mean cancellous bone intensity of the above vertebra.

### Descriptive statistics of the MR intensities histogram

The Gaussian distribution of the MR intensities histogram for the IVD, NP and AF was analyzed using the following indices: the standard deviation, the standard error, the median value, the sum of squared values, the 75^th^ percentile, the maximal voxel count in an intensity interval, the Skewness (index on the symmetry of the Gaussian distribution of the histogram) and the Kolmogorov-Smirnov distance (maximum cumulative distance between the histogram distribution, and the Gaussian distribution of intensity data).

### Volume ratio between NP and full disc

The volume ratio was computed as the ratio between the number of voxels within the segmented NP 3D matrix and the number of those within the segmented IVD 3D matrix as described by Violas et al. [41].

### Distance between weighted and geometrical centers

**Figure 2:**
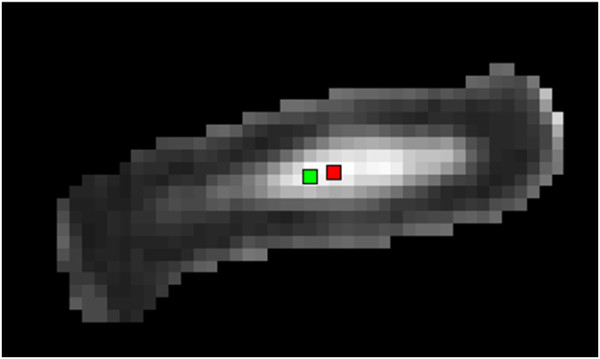
**2D projection of the 3D distance between the geometric center (G) and the MRI intensity weighted center (W) in a spondylolisthesis patient’s IVD** .

The center of intensity (W) was adapted from Périé et al. [42]. It was defined as a tridimensional point which position was weighted by the MR intensity in each IVD, NP and AF zones (Figure 2) and calculated as follow:

(1) $$W\left(x,y,z\right)=\frac{\sum_{i=1}^{n}I_{i}\left(x_{i},y_{i},z_{i}\right)P_{i}\left(x_{i},y_{i},z_{i}\right)}{\sum_{i=1}^{n}I_{i}\left(x_{i},y_{i},z_{i}\right)}$$

where I is the MR signal intensity at the space position P described using x, y, and z coordinates in the local coordinate system of each disc and n is the total number of points in the segmentation.

The normalized distance (d) between the center of intensity W and the geometric center (G) of the IVD, which allows minimizing the effects of the variable IVD sizes and different local coordinate systems of each disc between subjects on the result, was calculated as follow:

(2) $$d=\frac{\left|\text{W}\left(\text{x,y,z}\right)-\text{G}\left(\text{x,y,z}\right)\right|}{\text{N}}$$

where N is the total number of voxels in the studied zone. This index represents an undirected translation in the IVD morphology from an ideally symmetric disc to a pathologic disc.

### Statistical analysis between groups

Statistical analyses were performed using SIGMAPLOT 11.0 (Systat software Inc., San Jose, CA, USA). Differences between the scoliosis, spondylolisthesis and control groups were investigated using a one-way ANOVA for normally distributed, equal variance data. Kruskal-Wallis ANOVA and Dunn’s method of comparison were used for non-normal distributions. Differences between severity (low and high) and pathologic groups (Scoliosis and spondylolisthesis) were investigated using a two-way ANOVA for normally distributed, equal variance data. ANOVA on ranks with manual rank ordering was used for non-normal distributions. The distribution of the morphological parameters within the groups (Table 1) was unbiased and considered to have a negligible effect on the data.

## Results

### CSF normalized histograms of IVD

**Figure 3:**
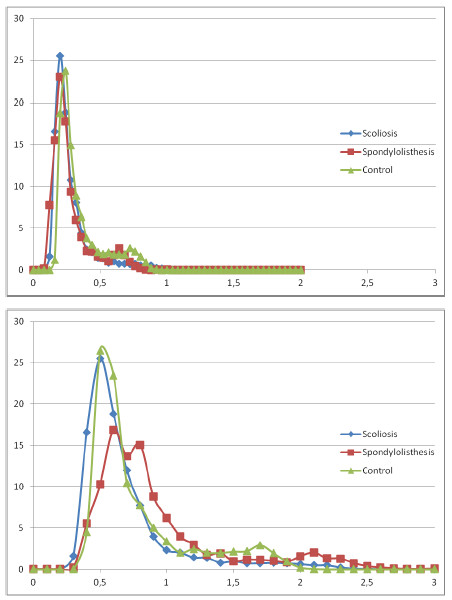
**Comparative MRI signal intensity histogram in the IVD between pathologies from normalized data using the average value of the CSF intensity (a) and the average value of the cancellous bone (b).** The x axis represents the gray level and the Y axis the number of pixels.

In the AF, significant differences were found in the standard deviation, standard error, 75^th^ percentile and Kolmogorov-Smirnov distance between the control group and the groups with spinal deformity (Table 2). The Kolmogorov-Smirnov distance was significantly larger in low severity than in high severity groups. In the NP, significant differences were found in the standard deviation, standard error and Kolmogorov-Smirnov distance between control and scoliotic subjects. However, no significant differences were observed between the scoliosis and spondylisthesis groups. In the IVD, no significant differences were observed for all parameters between all groups, as illustrated by the similarity between the histograms (Figure 3a).

**Table 2:** Significant differences found on the descriptive statistics of the histogram between pathology and severity groups

	**Region of interest**	**Standard deviation**	**Standard error**	**Kolmogorov-Smirnov distance**	**75**^**th**^**percentile**	**Median**	**Maximal voxel count in an intensity interval**	**Sum of squared values**	**Skewness**
***Control/Scoliosis***									
CSF normalization	AF	**	**	**	**		**		
	NP	*	*	**					
Bone normalization	AF	*	*	**	**	**	**		
	NP			*					
***Control/Spondylolisthesis***									
CSF normalization	AF	**	**	**	**		**		
Bone normalization	AF	*	*	**	**	**	**		
***Scoliosis/Spondylolisthesis***									
Bone normalization	IVD			*					
***Severity low/high***									
CSF normalization	AF			*					
Bone normalization	IVD	*	*					*	*
	AF								*

Results were considered significant (*) for p < 0.05 and highly significant (**) for p ≤ 0.001.

### Bone normalized histograms of the IVD

In the AF, significant differences were found in the standard deviation, standard error, maximal voxel count in an intensity interval, median, 75^th^ percentile and Kolmogorov-Smirnov distance between the control and the groups with spinal deformity (Table 2). Significant differences were also noted in the skewness between the severity groups. In the NP, significant differences were observed in the Kolmogorov-Smirnov distance between the control and the scoliotic groups. Similarly, in the IVD, the Kolmogorov-Smirnov distance was significantly higher for scoliosis than spondylolisthesis subjects, as illustrated by a different histogram shape for spondylolisthesis than for scoliosis or control (Figure 3b). Significant differences were also found in the standard deviation, standard error, skewness and sum of squared values between severity groups.

### Mean bone and CSF intensities

The mean bone intensity was not influenced by neither the pathology (p = 0.19) nor the severity (p = 0.72). On the contrary, the mean CSF intensity was highly influenced by both the pathology (p = 0.02) and the severity (p = 0.04). The CSF being constant between subjects, these signal changes are only due to the gain of the MR system between acquisitions. Thus the gain of the MR system is influenced by both the pathology and the severity. However, the ratio of the bone mean intensity divided by the CSF mean intensity was not linked to the pathology (p = 0.42) nor its severity (p = 0.1). The coupled effect of the MR system gain and vertebral bone changes are not influenced by the pathology nor its severity.

### Volume ratio between NP and full disc

A significant decrease was observed between control and pathologic groups (p ≤ 0.001, Table 3). However, no significant differences were observed between the scoliosis and the spondylisthesis groups, and between the severity groups.

**Table 3:** Volume ratio (%) between the NP and IVD for each pathology and severity group

**Group**	**Severity**	**Mean value**	**Standard deviation**
Scoliosis	Low	18.9%	3.4%
	High	19.9%	5.0%
Spondylolisthesis	Low	22.2%	3.6%
	High	18.0%	4.8%
Control		27.0%	6.3%

### Distance between weighted and geometrical centers

In the AF, NP and IVD, a significant increase of 50% (Table 4) was found (p ≤ 0.001, Table 5) between the control and the scoliosis groups. Highly significant differences were also detected (p ≤ 0.001, Table 5) between the scoliosis and the spondylolisthesis groups. Similarly, significant differences was found (p = 0.05, Table 5) between severity groups in the NP zone. However, no significant differences were found between the control and the spondylolisthesis groups in any zone.

**Table 4:** Normalized distance between weighted and geometrical centers for each pathology and severity group

**Pathology**	**Severity**	**Mean value**	**Std**
***IVD***			
Scoliosis	Low	0.50	0.28
	High	0.50	0.17
Spondylolisthesis	Low	0.12	0.09
	High	0.16	0.10
Control		0.22	0.11
***AF***			
Scoliosis	Low	0.52	0.28
	High	0.50	0.22
Spondylolisthesis	Low	0.28	0.12
	High	0.35	0.28
Control		0.20	0.06
***NP***			
Scoliosis	Low	1.86	1.43
	High	2.01	1.12
Spondylolisthesis	Low	0.80	0.56
	High	1.41	0.91
Control		0.95	0.52

**Table 5:** Significant differences on the distance between weighted and geometrical centers and on the volume ratio between pathology and severity groups

	**Region of interest**	**Significance**
***Control vs. Scoliosis***		
Distance between weighted and geometrical centers	IVD	**
	AF	**
	NP	**
Volume Ratio		**
***Control vs. Spondylolisthesis***		
Volume Ratio		**
***Scoliosis vs. Spondylolisthesis***		
Distance between weighted and geometrical centers	IVD	**
	AF	**
	NP	**
***Severity Low vs. High***		
Distance between weighted and geometrical centers	NP	*

Results were considered significant (*) for p < 0.05 and highly significant (**) for p ≤ 0.001.

## Discussion

The histograms of MR intensities within the intervertebral discs were analysed for the first time using descriptive statistics. Our hypothesis was validated as the results revealed a variation of the MR signal intensity within T2-weighted images of IVDs, which was different between spine pathologies and their severities. The AMRSID method and the new parameters proposed to analyse the MR signal within the IVD has the potential to provide essential information on spine pathologies that could not be appreciated by direct visual observation as currently performed in clinical routine.

### Descriptive statistics of normalized histograms

The results highlighted the following links between MR signal intensity and morphologic variations of the IVD. The standard deviation represents the width of the Gaussian peak and rises with increasing severity, which represented a decrease for the transition sharpness between the AF and NP, and morphologically the degeneration of the inner AF and NP. The sum of square showed the loss of the high white intensities values within the IVD and rose with increasing severity, which represented the loss of the healthy NP and inner-AF and thus was likely to reflect their degeneration. The Skewness detected the loss of inner AF intensities and rose with increasing severity, which represented the structural degeneration of the AF. The Kolmogorov-Smirnov distance detected the original slope of the curve as well as the loss of high AF and IVD intensities. It was greater for scoliotic than for spondylolisthesis patients and decreases with increasing severity. It gave essential information on the structural degeneration of the IVD with a loss of the original morphologic equilibrium. The maximum, the median and the 75^th^ percentile likely illustrated the homogeneity of the IVD tissues given by the concentration of the total MR intensity data around a few number of intensities. The more homogeneous the IVD, the more degenerated it was. This degeneration reflected a loss of the collagen matrix integrity in the NP and a diffusion of the molecules such as proteoglycans, leading to a loss of osmotic pressure and of disc hydration. The standard error represented the variation of the mean value between the perfect Gaussian and the obtained distribution and rose with increasing severities, which represented a decrease of the IVD’s hydration on T2-weighted images.

### Volume ratio and weighted center

The results were consistent with those in the literature with a mean volume ratio equal to 24% for scoliotic discs [41]. This index was found higher for control than for pathologic groups, which was expected since it detected the loss of the NP volume within the pathologic discs. The distance between the weighted and geometrical centers was only analysed in 2D by Perie et al. [6,26,42]. Our 3D index, which showed significant differences due to the pathology and severity, related the geometric intensity distribution variations and thus expressed structural and composition changes in the IVD, AF and NP. It detected the asymmetry in the IVD and described the tridimensional geometry of the disc degeneration. A clinical longitudinal follow-up of these indices would lead to a better understanding of the various IVD degeneration processes caused by spinal deformities. It is believed to have potential in providing critical information on the evolution of the spinal pathologies, and of the treatment.

### Bone and CSF normalizations

Normalizations were based on CSF and bone tissues MR signal intensity. Because of its constant composition between patients, the CSF normalization suppresses the acquisition gain from the signal intensity. Because bone tissues are known to remodel with spine pathologies [43,44], the bone normalization evaluated bone health and suppressed the acquisition gain at the same time. Based on the highly significant differences between groups from this study, we concluded that CSF normalization was able to detect differences between healthy and pathologic IVD whereas bone normalization detected more differences between the severities of these pathologies. The bone normalization was able to reflect both the bone and IVD health. This particularity allowed finding differences between the two severity groups.

### Limitations of the study

Each patient group was created to separate the surgical cases from the non-surgical cases. However, the distribution of age, height, weight, body mass index and MRI acquisition time of the patients in each group was heterogeneous. Such parameters are known to influence the MR signal intensity because of physiologic variations during growth peak and diurnal changes within the IVD [38,45]. As no significant differences in the distribution of these parameters were found between each group, the randomization was assumed and their effect was neglected.

The control cases are not from normal subjects but from patients with a herniated IVD. Because the biomechanical forces that are responsible in the pathophysiology of the IVD disease are usually exerting their effect over several levels, we chose a healthy disc three levels away from the herniated IVD, and we verified with a radiologist that the chosen IVD did not have abnormal MR signal distribution.

Segmentations of the IVD, NP and AF zones were processed semi-automatically, including the user’s perception. Thus we studied the sensitivity of the method to the segmentation process and data normalization [46]. The repetition of the semi-automatic segmentation by a same operator did not influence the quality of the contour or our new MR distribution parameters while the skills of the operator influenced only the MR distribution parameters. The instructions given prior to the segmentation influenced both the quality of the contour and the MR distribution parameters. Bone normalization produces an index which jointly highlights IVD and bone health, whereas CSF normalization only suppresses the effect of the acquisition gain. Segmentations used in this study provided reliable analysis results.

The segmentation is the longest process in our data analysis as it requires a manual intervention of the operator and takes about fifteen minutes per disc. An automatic process would decrease drastically this segmentation time. Some attempts to perform automatic segmentation of the IVD were proposed on T1-weighted images [47,48], but not for T2-weighted images in which there is not enough contrast between bone and annulus fibrosus.

MRI studies are realized in supine position and not in standing posture. However, our team demonstrated that the MR parameters of IVDs are not sensitive to compression (up to 40% deformation) neither to the partial confined relaxation that followed the compression [49].

### Clinical applications

X-ray films do not allow the analysis of the IVD, except its height that shows late stages of degeneration. The AMRSID method showed promising results that suggested the potential to change the prognosis or treatment options of scoliosis and spondylolisthesis. In AIS, the quantitative analysis of the apical disc may help predict curve progression in primary or secondary curves by indicating the level of IVD abnormalities and degeneration. However, no study has already correlated the IVD changes to the prognosis or the rate of progression of the AIS. To that end, longitudinal follow-up studies associated to the AMRSID method are necessary, in which the comparison of the MR signal distribution patterns will allow the definition of predictive factors of scoliosis progression using contingency tables, relative risk and percentage attributable risk factors. In L5-S1 spondylolisthesis, the AMRSID method may help surgical decision making by revealing evidence of disc degeneration above the affected level, and help surgeons to decide if this adjacent level should or should not be included in the fusion. Consequently, the clinical practice could be modified by the inclusion of a MRI acquisition associated to the AMRSID method for the diagnosis. Moreover, the treatment planning could be monitored in part by the results of the AMRSID method.

The application of the AMRSID method to elderly subjects needs to consider the IVD aging as a factor influencing the MR signal. Thus, a similar study has to be performed to verify that the distribution of the MRI signal intensity within the IVD in T2-weighted images depends on the spinal pathology, its severity and the aging degradation.

Images with enhanced resolution could lead to the discovery of more significant indices describing scoliosis and spondylolisthesis. Moreover, multi-parametric MRI is known to provide information on mechanical, structural and biochemical properties of the IVD [50-53] and could lead to new indices reflecting more aspects of the changes induced in the IVD by spine pathologies. Proton MR-spectroscopy, which evaluates the relative concentration of metabolites in tissues, might be used to assess IVD degeneration and proteoglycan content [54,55].

## Conclusions

This study proves for the first time that changes in the intervertebral disc, non appreciated by direct visual observation as currently performed in clinical routine, can be detected from specific indices describing the distribution of the signal intensity on sagittal T2-weighted MR images of the spine. Moreover, these indices are able to discriminate between the spinal deformities and their severities, and provide essential information on the composition and structure of the discs whatever the spine deformity considered. The AMRSID method has the potential to complement the current diagnostic tools available in clinics to improve the diagnostic with earlier biomarkers, the prognosis of evolution and the treatment options of scoliosis and spondylolisthesis. However, before being used in clinics, the resolution of the MR images has to be increased during the acquisition or during the post-treatment, an automatic segmentation process of the IVD has to be developed to reduce the data treatment duration, and longitudinal studies have to be performed to investigate the relationship between the IVD degenerescence and the evolution of scoliosis and spondylolisthesis.

## Competing interests

The authors declare that they have no competing interests.

## Authors' contributions

JG carried out the developments, all the data analysis, discussed the results and drafted the manuscript. CE, SP and HL participated to the results discussion and corrected the manuscript. DP proposed the design of the study, participated to the results discussion and the draft of the manuscript, and made the revisions of the manuscript. All authors read and approved the final manuscript.

## Pre-publication history

The pre-publication history for this paper can be accessed here:

http://www.biomedcentral.com/1471-2474/13/239/prepub
